# Advances in Resting State Neuroimaging of Mild Cognitive Impairment

**DOI:** 10.3389/fpsyt.2018.00671

**Published:** 2018-12-06

**Authors:** Li Lin, Guoqiang Xing, Ying Han

**Affiliations:** ^1^Department of Neurology, XuanWu Hospital of Capital Medical University, Beijing, China; ^2^Department of Imaging & Imaging Institute of Rehabilitation and Development of Brain Function, The Second Clinical Institute of North Sichuan Medical College, Nanchong Central Hospital, Nanchong, China; ^3^Beijing Institute of Geriatrics, Beijing, China; ^4^National Clinical Research Center for Geriatric Disorders, Beijing, China; ^5^Center of Alzheimer's Disease, Beijing Institute for Brain Disorders, Beijing, China

**Keywords:** fMRI, resting state, mild cognitive impairment, Alzheimer's disease, functional connectivity

## Abstract

The rapidly increasing number of patients with Alzheimer's disease (AD) worldwide has become a major public concern. Mild cognitive impairment (MCI), characterized with accelerated memory decline than normal aging, is a stage between cognitively unimpaired and dementia. Identification of MCI in the Alzheimer's continuum from normal aging, is important for early diagnosis and improved intervention of AD. The imaging technique has been extensively used for diagnose and understanding the mechanisms of MCI. Firstly, we review the recent progresses in the research framework of MCI depending on the clinical and/or biomarker findings. Secondly, we cover studies that use of rs-fMRI (resting state functional magnetic resonance imaging) for the brain activities and functional connectivity between normal aging and MCI. Other methodologies and multi-modal studies for investigating the mechanism and early diagnosis of MCI are also discussed. Finally, we discuss how genetic and environmental factors such as education could interact with in MCI. Overall, MCI is a heterogeneous state and employing resting state neuroimaging with other AD biomarker approaches will be able to target in the more precise population and AD-related pathology process.

## Introduction

Alzheimer's disease (AD) is the most common cause of dementia in older adults worldwide. There will be one new case of dementia every 3 s. By 2030, the number of people with the disease is expected to rise to more than 70 million (1). The burden of AD patients on themselves, their families, and society has risen considerably and attracted significant attention. Although much attention has been paid to AD, no effective treatment for AD are available to date. Nowadays, drugs may produce some short-term improvement in cognitive function but they cannot slow down or stop the progression of the pathological damages if one is currently diagnosed AD dementia which is in the late stage of the disease. Despite of this, diagnosis at the early phase of the disease, like preclinical stage, or prodromal stage, may represent a good opportunity for interventions (2).

To better diagnose and define clinical stages of AD, two major diagnostic criteria, the National Institute of Aging and Alzheimer's Association (NIA-AA) criteria and the International Working Group(IWG) criteria, for AD were proposed (3–5). The NIA-AA criteria describes an entire clinical spectrum and the related biomarkers of AD (4, 5). Mild cognitive impairment (MCI) is an intermediate stage between cognitively unimpaired and dementia. Fifteen to twenty percentage of people aged 65 or older have MCI and these patients have a high probability of converting to AD, at an average of 32% in 5 years (6–8). Identifying these individuals with MCI could be an effective strategy for early diagnosis and treatments to delay and/or halt AD progression toward an irreversible full brain damage (9).

Toward greater understanding of the mechanisms of AD, not only clinical or cognitive tests, but also biological methods have to been used. Neuroimaging studies have recently begun to illuminate structural and functional brain abnormality in early AD and even constitute a vital part of the research framework about AD (5). Resting-state functional magnetic resonance imaging (rs-fMRI), which measures the spontaneous fluctuations of blood oxygenation level-dependent (BOLD) signals in different brain regions without tasks, has been widely used to investigate different diseases, such as psychosis (10, 11), depression (12), Huntington's disease (13), stroke (14), and AD (15, 16). During rs-fMRI, participants rest with their eyes closed or focus on a visual fixation while being examined. This is more convenient and less taxing than task-based fMRI or neuropsychological tests for patients with cognitive impairment. Functional connectivity within and between different brain regions can be assessed, and intrinsic brain networks such as the default mode, executive control, visual, salience, and auditory networks can be identified by analyzing BOLD signals (17). These studies suggest that aberrant regional spontaneous fluctuations of BOLD (18, 19), functional connectivity (20), and widespread alterations in functional brain network architecture (21, 22) could all occur early in AD pathophysiology. Rs-fMRI can also contribute to evaluating the different treatment and detect even subtle changes after a short period of treatment (23). Overall, resting state neuroimaging and other methodologies may provide useful biomarkers for early diagnosis of AD and understanding the underlying mechanism (24).

In this paper, we review recent progresses of MCI criteria and nomenclature, resting state neuroimaging studies on brain functional activity in patients with MCI, current knowledge of the influences of genetic and educational factors on MCI and the work on diagnosing and classifying MCI. In this review, we focus on the fMRI-based studies and amnestic MCI, but we mention other approaches and MCI with AD biomarkers/pathologic change as well.

## Evolution of MCI

MCI was first proposed by Petersen et al. (25) and the diagnosis was mostly depended on clinical or cognitive appearance. After that, many researchers have investigated and expanded the nomenclature. A few criteria (4, 26–28) were derived from these studies and MCI was noticed to be heterogeneous and unstable. MCI could be one domain affected or multiple-domain affected and it could be caused by Alzheimer's disease, vascular dementia, depression, or other medical conditions (29). Without general or recommended biomarkers, in a long period, researchers applied different diagnostic criteria to define MCI. The core definitions of amnestic MCI in early criteria are the following: (1) not normal for age; (2) cognitive decline; (3) essentially normal functional activities; (4) not demented; (5) memory impaired. In 2010s, NIA-AA (4) and IWG (3) became more aware of the importance of biomarkers and put more emphasis on A β, tau, and other AD related biomarkers. NIA-AA proposed a new research framework toward AD in 2018, and MCI could either be a syndromal cognitive stage between cognitive normal and dementia which was consistent with the past criteria or be detailed refined according to biomarker profile and cognitive appearance (5). With this new research framework, researchers should enable to target more precise AD-related MCI, and enhance efforts to understand the pathology process of AD and different etiology of dementia. However, the applications of core AD-related biomarkers are expensive, not necessary to apply for every MCI study. On the other hand, we should develop a less-expensive and credible biomarker to investigate the MCI, as the population of MCI is even larger than AD. The criteria of MCI were summarized in Table 1.

**Table 1:** MCI diagnosis criteria used by neuroimaging studies.

**Items of recent criteria**	**Subjective memory complaint**	**Corroborated by an informant**	**Objective cognitive impairment (especially memory)**	**Normal activities of daily living**	**Not demented and not normal**	**Subcategorization: single / multiple domain**	**Biomarkers**	**A β (PET or CSF)**	**Tau (PET or CSF)**	**Neuronal injury (FDG, sMRI, fMRI)**	**Nomenclature**
Petersen et al. (25)	Yes			Yes	Yes						MCI
Petersen et al. (27)	Yes	Yes	Yes	Yes	Yes						aMCI
Winblad et al. (28); Gauthier et al. (26); Petersen and Negash (29)	Yes	Yes	Yes	Yes	Yes	Yes					aMCI
ADNI	Yes	Yes	Yes	Yes	Yes	Yes	Yes				aMCI
Dubois and Albert (30)/ IWG2007 (31)	Yes		Yes (hippocampal type)	Yes	Yes		Yes	/	/	/	Prodromal AD or MCI of Alzheimer-type
NIA-AA2011 (4)	Yes	Yes	Yes	Yes	Yes	Yes	Yes	Conflicting/ in determinant/ untested	Conflicting/ in determinant/ untested		MCI-core clinical criteria
								Positive	Untested		MCI due to AD—intermidiate likelihood
								Untested	Positive		MCI due to AD—intermidiate likelihood
								Positive	Positive		MCI due to AD—high likelihood
NIA-AA2018 (5)		Yes	Yes	Yes	Yes		Yes	Positive	Negative	Negative	Alzheimer's pathologic change with MCI
								Positive	Positive	Positive /negative	Alzheimer's disease with MCI (prodromal AD)
								Positive	Negative	Positive	Alzheimer's and concomitant suspected non Alzheimer's pathologic change with MCI
								Negative	Positive/ negative	Positive/ negative	Non-Alzheimer's pathologic change with MCI

## Cognitively Unimpaired Aging

To investigate the aberrant brain activity in cognitive impaired old people, changes of brain structure, and function associated with aging should be firstly documented. Raichle (17), Salvador et al. (32), and Achard et al. (33) have demonstrated a sensible, symmetrical architecture of the human brain which could be characterized with a few networks and organized with a small-world network topology. The functional connectivity and structure alteration of brain were associated with memory and aging. Specifically, Ward et al. (34) reported that default mode network (DMN) functional connectivity and hippocampus volume were associated with memory among old individuals. Inter-network functional activity also changed as people aged. Anticorrelated activity between DMN and dorsal attention network was found significantly decreased with age (35). The degrees of functional alteration were different among different brain regions and cognitively normal individuals.

Importantly, AD biomarkers, like amyloid burden, mediate the relationship between age and brain function. Using *in vivo* amyloid imaging and fMRI, both Hedden et al. (36) and Sperling et al. (37) found aberrant DMN activity in cognitively unimpaired aging with amyloid positive. The patterns of disruption of functional activity were linked to amyloid pathology. Cognitively unimpaired aging with high amyloid burden displayed more reduced functional correlations within posterior cingulate cortex (PCC) and other regions related to memory encoding. Especially, these alterations were stable after controlling for age and structural atrophy which suggested clinically normal older people with disrupted functional activity, particularly with biomarkers of AD, were the susceptible population of AD. Notably, the relationship between amyloid burden and functional connectivity is complex and may be biphasic or multi-phasic changes across the longitudinal process of Alzheimer's continuum. By way of example, Lim et al. (38) reported that greater DMN functional connectivity in cognitively unimpaired aging with positive amyloid burden compared with those with negative amyloid burden. For defending the hazards of amyloid pathology, the brain may develop a response with compensatory higher functional connectivity in some brain regions (39).

## Mild Cognitive Impairment

Recent studies have demonstrated distinct intrinsic functional brain network architectures in patients with various neurodegenerative diseases, and with each disease having a distinct abnormal brain activity pattern (40, 41). The MCI patients were characterized with disruption of functional connectivity within DMN (19, 42, 43) which involved the ventral medial prefrontal cortex, dorsal medial prefrontal cortex, PCC, adjacent precuneus, and lateral parietal cortex, indicating brain activity as a potential target of diagnose. Regional homogeneity (ReHo) (44), amplitude of low-frequency fluctuation (ALFF) (45), functional connectivity analysis as well as graph theory have been used to investigate the underlying pathological process of MCI.

### ReHo and ALFF

**Figure 1:**
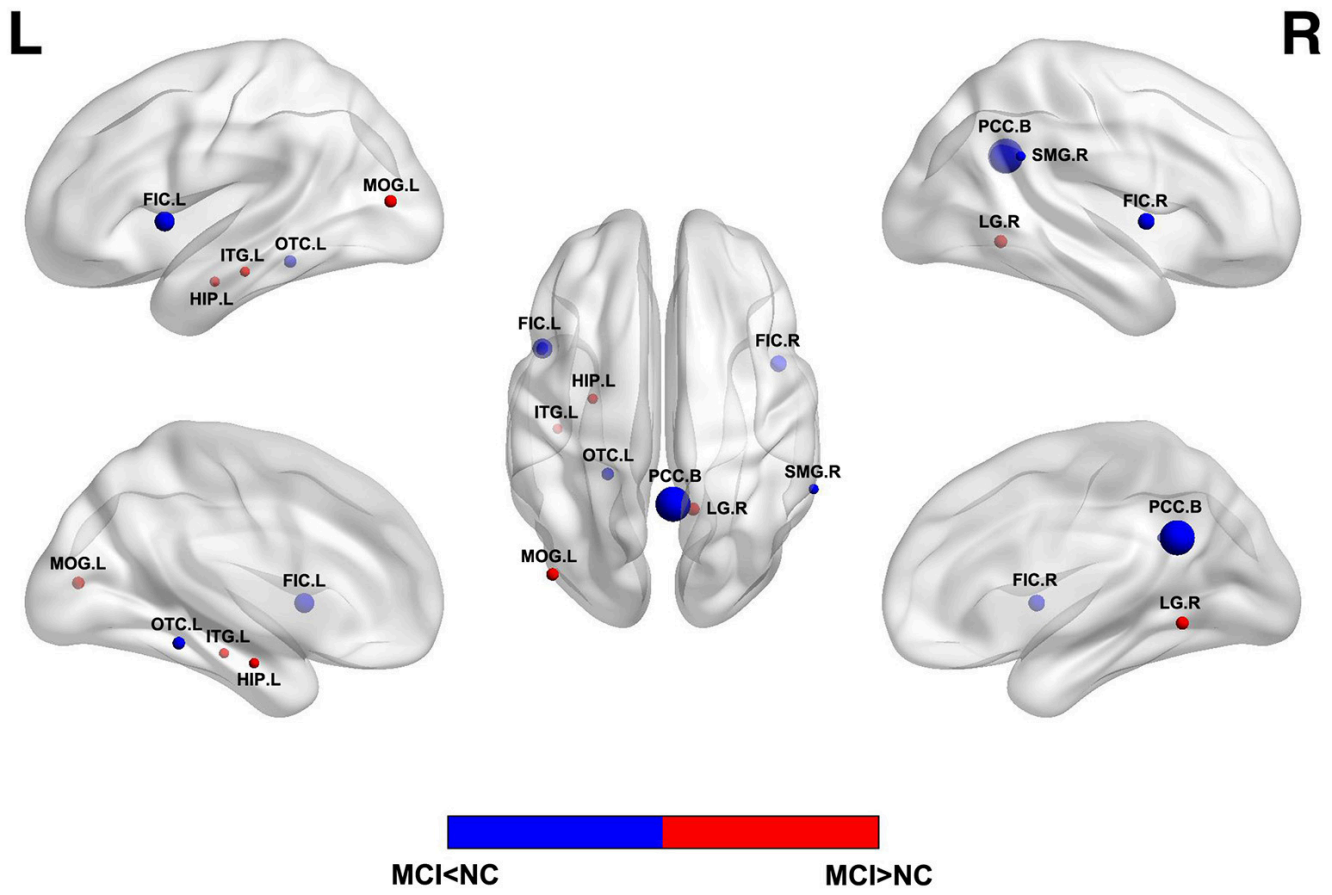
Abnormal regional functional activity in MCI patients compared with normal controls. Blue = lower functional activity in MCI patients vs. normal controls, and red = higher functional activity in MCI patients vs. normal controls. The sizes of balls represent the relative areas of abnormality in these brain regions. L, left; R, right; B, both hemispheres; FIC, frontoinsular cortex; OTC, occipitotemporal cortex; SMG, supramarginal gyrus; LG, lingual gyrus; MOG, middle occipital gyrus; HIP, hippocampus; ITG, inferior temporal gyrus.

ReHo assumes that a given voxel is temporally similar to that of its neighbors and voxels within a functional brain area are more temporally homogeneous when this is involved in a specific condition. ALFF is found highly synchronous among functional brain systems in normal subjects and less synchronous in patients with mental diseases. Changes in the resting state brain activity of MCI have been evaluated by ReHo or ALFF of BOLD signals, as illustrated in Figure 1. Aberrant ReHo was found prefrontal cortex, bilateral posterior cingulate gyrus/precuneus and inferior parietal lobule in patients with MCI, compared with normal aging (19, 46). Similar abnormal functional patterns were reported in ALFF studies of MCI (18) and these patterns exhibited different spatial patterns depending on various frequency bands (42). Both increased and decreased activity could coexist in MCI. The decreased activity in the cuneus/precuneus was found to be associated with reduced memory performance (18) and the increased activity could be a compensation for damage by recruitment of other regions. However, as MCI is a heterogeneous state, AD biomarker related approaches and detailed cognitive examinations may bear more fruit. A recent study combing rs-fMRI and CSF showed the ReHo value was associated with A β level in superior temporal gyrus in single domain MCI while the multiple-domain MCI exhibited a more complex pattern of pathology and functional activity (47).

### Functional Connectivity

**Figure 2:**
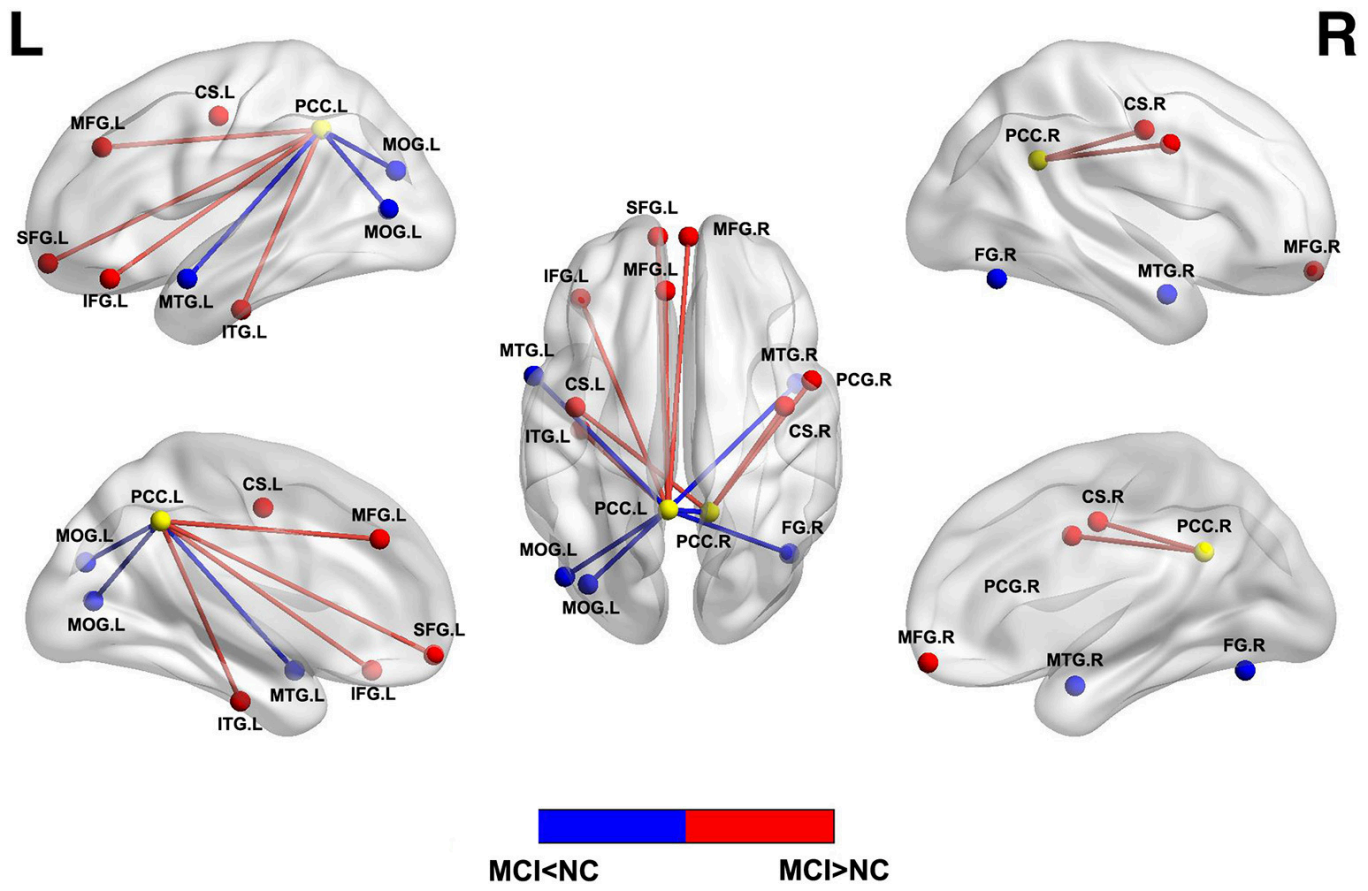
Abnormal functional connectivity between PCC and other brain regions in MCI patients compared with normal controls. Blue = lower functional connectivity in MCI patients vs. normal controls, and red = higher functional connectivity in MCI patients vs. normal controls. L, left; R, right; MOG, middle occipital gyrus; MTG, middle temporal gyrus; FG, fusiform gyrus; SFG, superior frontal gyrus; MFG, medial frontal gyrus; IFG, inferior frontal gyrus; ITG, inferior temporal gyrus; CS, central sulcus; PCG, precentral gyrus.

Resting state functional connectivity describes temporal correlations of BOLD signal between different brain regions/voxels and is the basis of graph theory analysis. Independent component analysis (ICA) and seed-based analysis are the two main methods to analyze the functional connectivity. Early rs-fMRI studies have shown impaired functional connectivity within DMN or between different networks in patients with MCI (22, 48). Subsequent studies revealed decreased functional activity in the DMN regions and increased functional activity in the frontal cortex and other regions (Figure 2) (48–50), which indicated that the disrupted and compensatory patterns were general in MCI patients and could be detected by different resting state analysis. Similarly, considering the methodological differences or clinical heterogeneity or biphasic changes across the longitudinal process, the degree of alteration of functional connectivity in the hippocampus and PCC was consistent with poor performance of neuropsychological tests in MCI, indicating the potential of functional connectivity as a biomarker of cognitive preditor (51–53).

In addition to the alteration of DMN in MCI, which has been widely reported, altered functional connectivity within, and/or between different other brain networks such as the executive control, salience, dorsal attention, and sensory-motor networks may also occur in MCI (54–56). For instance, Brier et al. (56) recruited 510 participants with CDR (clinical dementia rating) ranging from 0 to 1 to investigate five functional brain networks through rs-fMRI. They found decreased connectivity within all networks and decreased anti-correlations between these networks as CDR increased. Specifically, some networks were preferentially affected at certain CDR stages. Similarly, Esposito et al. (35) suggested that anti-correlation between DMN and DAN decreased with age, but such reduction was more significant in patients with MCI. Furthermore, with regard to whether such functional changes were generated by aging or pathology process of AD, Brier et al. (57) examined two groups cognitively normal people with or without AD-related CSF biomarkers. Their results suggested that AD pathology accounted for a large portion of the alterations of functional connectivity. It is still not fully understood how and when elevated AD pathology affects functional connectivity, but these studies indicate the possibility of finding out the relationship between functional connectivity and AD pathology, which could help to establish a safer and/or less-expensive approach for targeting pre-dementia population.

### Graph Theory

Graph theory has been widely used to analyze the organization of the societies, information networks, and internet, but has rather limited use in neuroscience until now. Recent studies have applied this approach to investigate the brain's topological organization (32, 33) that revealed disrupted topological organization of the whole brain in MCI (58, 59). Based on rs-fMRI, patients with MCI showed decreased overall global functional connectivity of brain connectome and the hubs and important connections between DMN and other functional systems were impaired within MCI patients (58). Such disruption could be partially verified or caused by structural loss of neural fibers, such as corpus callosum, as detected by DTI (60). In addition, as the graph theory analysis assesses the brain activity as a whole, it results in different and new insights of understanding the mechanism of MCI. Zhang et al. explored the connectome in MCI with two dimensions. They reported that both local and remote connectivity dysfunctions were detected in MCI with rs-fMRI while episodic memory performance in MCI was only associated with remote connectivity within DMN. Liu et al. (61) found abnormal rightward laterality in MCI compared with normal controls, which indicated a similar compensatory mechanism detected with above rs-fMRI analysis but displayed in a different appearance. Further, because these changes in functional connectome were correlated with patients' memory performance, graph theory analysis could also act as a method of differentiating individuals with MCI from normal aging (58).

### Multi-Modal Imaging Study

Although rs-fMRI is one of the convenient and noninvasive imaging modality to study pathophysiology of MCI, combination with other techniques can investigate both abnormal structural and functional features as well as their relations in MCI, improve the quality of related clinical trial and reduce requirement of sample sizes (62, 63). Multi-modal imaging studies have confirmed correlations between structural and functional alterations (63, 64). Collecting gray matter volumes and functional connectivity information simultaneously could serve as a better indicator for predicting the cognitive deficits in MCI (65). In a recent multi-modal study, a novel PET/fMRI scanner was applied to normal aging, patients with MCI and AD to evaluate the resting-state brain glucose metabolism and brain functional activity (64). The metrics [ReHo, fractional ALFF and group ICA with dual regression(gICA-DR)] of rs-fMRI were showed to be correlated with glucose metabolism among all three groups, but the degree of correlation reduced 17% in MCI/AD groups. With the progression of the disease or higher amyloid levels, MCI patients showed severer hippocampus atrophy, reduced structural connectivity of the corpus callosum, and lower functional connectivity between hippocampus and precuneus and higher hippocampal metabolism and amplitude of delta rhythms at rest (66, 67). Although criteria and investigators have paid more attention on AD biomarker profile in the last decade, the number of multi-modal studies and participants is still small compared with that of single modal studies. To address this, combing rs-fMRI with AD biomarker examinations and multi-center collaborations should be more stressed.

## Early Diagnosis and Conversion of MCI

### Early Diagnosis of MCI

Early diagnosis of MCI is important for timely and potentially successful therapeutic intervention. Although substantial research has been dedicated in the last decades, identification of patients with MCI who are at risk of AD is still a challenge. Resting state neuroimaging is an efficient and powerful technique that facilitates the discovery of abnormalities in MCI patients for the consistency among amounts of studies. Various data processing methods have been used to diagnose or classify MCI based on rs-fMRI scans. Here, some main methods for achieving high diagnostic power for MCI are summarized in Table 2.

**Table 2:** Summary of studies using different models for early diagnosis of AD.

**Papers**	**MCI**	**Control**	**Imaging modalities**	**Data analysis**	**Acc. (%)**	**Sen. (%)**	**Spec. (%)**	**Auc. (%)**
Koch et al. (68)	17	21	fMRI	ICA & VOI-based time course	81.6	64.7	95.2	–
Qian et al. (69)	37	32	fMRI	CEEMD & SVM	93.3	–	–	94.1
Chen et al. (70)	15	20	fMRI	LSN & LDA	91.0	93.0	90.0	95.0
Khazaee et al. (71)	89	45	fMRI	SVM	72.0	84.9	61.5	–
Chen et al. (72)	29	30	fMRI	LASSO & SVM	88.1	86.2	90.0	93.0
Yu et al. (73)	50	49	fMRI	WSGR	84.8	86.8	72.1	86.8
Challis et al. (74)	50	39	fMRI	GP-LR	75.0	100%	50.0	70.0
Jie et al. (75)	99	50	fMRI	SVM	78	82	74	77
Wee et al. (76)	10	17	DTI & fMRI	SVM	96.3	100	94.1	95.3
Zhu et al. (77)	22	22	DTI & fMRI	CFS & SVM	95.4	95.0	95.9	–

*Acc., accuracy; Sen., sensitivity; Spec., specificity; ICA, independent component analysis; CEEMD, complementary ensemble empirical mode decomposition; LSN, large-scale network; LDA, linear discriminant analysis; SVM, support vector machine; LASSO, l_1_-norm regularized least squares regression; WSGR, weighted sparse group representation; GP-LR, Bayesian Gaussian process logistic regression; CFS, correlation-based feature selection*.

Most studies for classification of MCI through rs-fMRI were based on functional connectivity or network architecture features between normal individuals and patients. Some of them focused on the key network of MCI, distinguishing MCI from normal controls based on DMN brain activity of rs-fMRI (68). Others depended on the whole-brain activity (69), calculating functional connectivity between various regions (70), or using the graph theory metrics (71). Since the course of AD is continuous, the patients in AD continuum share many common features. On one hand, it's good for investigators to identify patients with MCI from normal aging; on the other hand, it becomes more difficult to differentiate between MCI and other stages. For example, one study analyzed DMN brain activity by using both volume of interest (VOI)-based signal time course and independent component analyses (ICA) for identifying patients with dementia (68). They found 82% patients with MCI contained the characteristics of AD patients. Chen et al. applied a large-scale network analysis to classify normal aging and patients with MCI and AD (70). The AUC analysis yielded 95% classification power, 93% sensitivity, and 90% specificity between patients with MCI and normal controls.

Great efforts have been made to improve the algorithms of classification (72, 73). Instead of simply correlating pairwise regional activities, recent studies used different approaches to construct correlation networks to simulate the actual biological networks of brain. By combining a graph theoretical approach with machine learning methods, Khazaee and colleagues achieved 88.4% classification accuracy of AD, MCI, and controls. Such approach used the optimal features extracted from graph measures by a support vector machine (SVM) (71). Chen et al. (72) proposed a model of constructing a high-order rs-fMRI functional connectivity network by grouping correlations for every pair of brain regions into different clusters, whose respective mean correlation time series were represented as high-order correlations among different brain regions. Other methods and models by analogy with biological connectivity in the human brain have also been proposed (73, 74). Moreover, a recent fMRI study not only investigated the temporal properties, but also focused on the temporal variability of functional connectivity between specific brain regions (75). As the development of 7T-MRI and improvement of algorithms of MRI, using spatio-temporal interaction patterns of brain activity to classify patients and normal controls and achieving more accuracy of classification will be easier in future.

To avoid the shortcomings of single neuroimaging modality and/or target the MCI patients in AD continuum, recent studies applied multi-modal combinations of sMRI, rs-fMRI, and DTI to classify MCI from controls. Wee et al. integrated diffusion tensor imaging (DTI) and rs-fMRI with multi-kernel SVM to improve the classification of AD, MCI, and normal aging (76). This approach yielded 95% AUC, 100% sensitivity, and 94% specificity between patients with MCI and normal controls. Another similar neuroimaging study also yielded high classification accuracy, at more than 95% (77). Thus, multimodal connectivity networks have yielded better results in identifying patients with MCI. Together, methods derived from the interactions among different functional networks or derived from multi-modal are better than that derived from a single network or one modal. However, we should bear in mind that many recent resting state studies used different criteria and approaches to diagnosis MCI, future research is required to replicate these findings and it is too soon to make a conclusion about the best algorithm of classification.

### Conversion of MCI

As a transient stage of cognitive stage, patients with MCI are likely to convert to AD, especially those with AD biomarkers. The rate of conversion of patients with MCI older than 65 years to AD when followed for 2 years was about 14.9% (9). Equally important to MCI's etiology, knowledge of the mechanism of conversion from MCI to AD can help to invent new treatments to slow or halt disease progression and modify available therapies. To date, neuroimaging studies have provided some clues of how MCI is converted to AD. Li et al. (78) tracked MCI patients who had neurocognitive tests and rs-fMRI before and after the conversion. The results showed a significantly lower functional connectivity between the left angular gyrus and middle occipital gyrus in the converters than non-converters. Another study reported both intra- and inter-network longitudinal disruptions in MCI, the decline of compensatory ability may act as the potential cause of conversion (79). Consistent with abnormal functional connectivity, study of the DTI imaging modality showed selective and progressive disruptions of structural connectivity in patients with MCI that could be used to separate converters from non-converters (80). As compared to the above studies which had similar small-moderate sample sizes, Buckley et al. (81) analyzed 237 clinically normal older adults' longitudinal data with amyloid imaging and rs-fMRI. They speculated that lower functional connectivity predicted more rapid cognitive decline and amyloid burden would accelerate this process. In addition, they suggested rs-fMRI may act as a predictor of early AD-related cognitive decline. In summary, there is no current consensus on how the MCI converts to next stage, and the approaches used exhibit vast heterogeneity.

## Influences of Genes and Education

### Influences of Genetics

Increasing evidence suggest that multiple factors, including diabetes, obesity, physical and mental inactivity, depression, smoking, low educational attainment, and genetics all may play a role in AD. Among them, genetic association with AD is strong (82). Previous studies have revealed that genes like ATP-binding cassette transporter A7 (ABCA7), clusterin (CLU), complement receptor 1 (CR1), apolipoprotein Eε4 (APOE4), phosphatidylinositol-binding clathrin assembly protein (PICALM), are associated with AD risk. The identification of these genetic loci and their mechanism of pathological function are crucial for understanding the etiology of AD and for early diagnosis. Combined neuroimaging-genetic studies can provide crucial evidence of genetic effects on brain functioning of MCI.

APOE4 is one of the major genetic risk factor for AD and is involved in lipid homeostasis in the brain that influences multiple neurophysiological pathways and MCI network dysfunction (83). Study of APOE4 showed a strong overlap in decreased functional connectivity between patients with MCI and APOE4 carriers with or without MCI (84). Only patients with MCI had decreased functional connectivity of prefrontal cortical areas compared with APOE4 carriers, suggesting that the etiology of MCI has multiple factors besides genetics. The results of another combined APOE4-rs-fMRI study showed that APOE4 may accelerate functional connectivity decline in related brain networks in patients with MCI (85). Conversely, APOE2 would produce age-dependent and divergent effect on functional connectivity (86).

Additionally, other metabolic pathways may also play a role in pathogenesis of MCI. CLU has been associated with brain atrophy (87). Bai et al. (88) correlated the results of CLU genotyping and rs-fMRI imaging in patients with MCI and normal controls, and found significant effects of the CLU CC genotype on cortical midline regions, especially task-positive networks in MCI. In a later study with similar methods, Bai et al. (89) found the promoter haplotypes of IL-10 may be associated with abnormal functional communications in the left hippocampal-frontoparietal cortex in MCI. Another inflammation-related gene, Interleukin 1 beta (IL-1β), was associated with abnormal ALFF in left parietal cortex, bilateral frontal cortex, and left occipital cortex in patients with MCI (90). PICALM gene polymorphisms were found to interact with the functional activity in the left middle temporal gyrus and left middle frontal gyrus (91). In addition, functional connectivity in the gene-related regions may be correlated with memory performance for a distinct genotype subgroup of patients with MCI (86, 91). Although it is clear for investigators that these genes may play a role in MCI pathogenesis, it's easy to be ignored the genes are originally functional in normal people. For instance, Seddighi et al. (92) suggested that low expression of SPARCL1 were associated with accelerated memory loss, reduced brain volumes, and decreased cerebral perfusion during aging. Future research should attempt to specify the interaction of different genetypes with pathology process in MCI with AD biomarkers.

### Influences of Cognitive Reserve

Recent work on the functional connectivity of the DMN and other related resting-state networks in patients with MCI suggested the coexistence of impairment and compensation. A factor called cognitive reserve (CR), which is often assessed via years of education and IQ, has attracted attention for its relationships to cognitive performance and level of brain damage. Studies suggested the attempts by the patients' brains to compensate for damage by using the existing faculties of functional cognitive brain regions. Neuroimaging studies show CR modulates functional connectivity in large-scale network in patients with MCI and that is correlated with memory scores (93–95). Serra et al. (93) reported that high CR in patients with MCI was associated with increased functional connectivity in a network of fronto-parietal nodes and decreased functional connectivity in a network of fronto-temporo-cerebellar nodes. A recent study of functional connectivity between the DMN and the DAN, which is associated with cognitive control and working memory, suggested that negative DMN-DAN correlation was associated with poorer memory performance in patients with MCI. However, after adjusting for the CR, this association could be compensated by a higher CR (95). Not surprisingly, rs-fMRI could conversely be used to predict the significance of CR in patients with MCI (96). However, since the cognitive function declines with age, whether the changes observed are due to the normal aging or the pathological process of AD should be considered.

## Future Work and Conclusion

As seen from the studies presented here, rs-fMRI has already contributed substantially to the field of MCI's research. Alteration of functional brain activity within DMN and between other networks have been detected with multiple analysis in cognitive impaired people, and such changes are accelerated by MCI related pathology process and other risk factors. As an intermediate stage of AD continuum, MCI patients reserve the capability of compensating the detriment of the disease. The possible compensatory mechanisms and how to differentiate the MCI due to AD from the pre-dementia are still a mystery. Longitudinal study design and improvement of data analysis would help to solve these problems. In future research, it will be important to determine which features in MCI are the signs of progression to AD. As imaging modalities improve, multi-center collaboration, and data exchanges expand, and as MCI criteria are more clearly delineated, we may expect further refinement in characterizing the association between MCI and brain and/or cognition based biomarkers.

## Author Contributions

LL, GX, and YH contributed conception and design of the study. LL wrote the first draft of the manuscript. All authors contributed to manuscript revision, read, and approved the submitted version.

### Conflict of Interest Statement

The authors declare that the research was conducted in the absence of any commercial or financial relationships that could be construed as a potential conflict of interest.
